# Associations of adverse childhood experiences with caries and toothbrushing in adolescents. The Young-HUNT4 Survey

**DOI:** 10.1186/s12903-023-03492-z

**Published:** 2023-10-14

**Authors:** Lena Myran, Abhijit Sen, Tiril Willumsen, Audun Havnen, Therese Kvist, Anne Rønneberg, Göran Dahllöf, Hedda Høvik

**Affiliations:** 1 Center for Oral Health Services and Research, Mid-Norway (TkMidt), Trondheim, Norway; 2 Department of Psychology, Faculty of Social and Educational Sciences, Norwegian University of Science and Technology (NTNU), Trondheim, Norway; 3 Department of Public Health and Nursing, Faculty of Medicine and Health Sciences, Norwegian University of Science and Technology (NTNU), Trondheim, Norway; 4 Department of Paediatric Dentistry, Behavioural Science and Forensic Dentistry, Institute of Clinical Dentistry, University of Oslo, Oslo, Norway; 5 Division of Psychiatry, Nidaros Community Mental Health Centre, St. Olavs University Hospital, Trondheim, Norway; 6 Division of Orthodontics and Pediatric Dentistry, Department of Dental Medicine, Karolinska Institutet, Stockholm, Sweden; 7 Center for Pediatric Oral Health Research, Stockholm, Sweden; 8 ME barnakutsjukvård, Astrid Lindgrens Children’s Hospital, Karolinska University Hospital, Stockholm, Sweden

**Keywords:** ACE, Adverse childhood experience, Adolescents, Caries, Cross-sectional, DMFT, Epidemiology, HUNT, Oral health, Toothbrushing

## Abstract

**Background:**

Adverse childhood experiences (ACEs) are associated with poor oral health. Using a life course theoretical framework, this study explored the associations of specific and cumulative ACEs with caries and toothbrushing frequency in a Norwegian adolescent population.

**Methods:**

Participants were adolescents (n = 6351) age 13–17 years from The Young-HUNT4 Survey. Clinical data were retrieved from dental health records. Oral health outcomes were toothbrushing frequency, dentine caries experience (Decayed, Missing, and Filled Teeth – DMFT), and enamel caries. ACE exposure variables were physical abuse, sexual abuse, witness to violence, parental separation/divorce, parental alcohol problems, and bully victimization. Negative binominal regression models (incident rate ratios, IRRs; 95% confidence intervals, CIs) were used to determine the associations of the various ACEs with caries; logistic regression analyses (odds ratios, ORs; 95% CIs) were used to estimate associations with toothbrushing frequency. Potential effect modification by age was assessed using likelihood ratio test.

**Results:**

Adolescents exposed to physical abuse by others, sexual abuse by peers, parental separation/divorce, bullying, or who had witnessed violence, were more likely to report non-daily toothbrushing compared with those with no exposure to the given ACEs. Each cumulative increase in ACE exposure was associated with a 30% higher likelihood of non-daily toothbrushing (OR 1.30, 95% CI 1.19–1.42). Similarly, increasing number of adversities were associated with both higher dentine caries experience (IRR 1.06, 95% CI 1.02–1.09) and higher enamel caries (IRR 1.07, 95% CI 1.03–1.11). This effect was modified by age (13–15 vs. 16–17 years) for dentine caries experience. Furthermore, there was evidence of effect modification by age with bully victimization for both toothbrushing frequency (P_interaction_ = 0.014) and dentine caries experience (P_interaction_ < 0.001). Specifically, bully victimization was associated with a higher likelihood of non-daily toothbrushing (OR 2.59, 95% CI 1.80–3.72) and higher dentine caries experience (IRR 1.30, 95% CI 1.14–1.50) among 16–17-year-olds.

**Conclusions:**

Several specific ACEs were associated with non-daily toothbrushing and a higher caries experience among Norwegian adolescents in the Young-HUNT4 Survey.

## Background

Adverse childhood experiences (ACEs) are defined as potentially traumatic events that occur before the age of 18 years [1] and accommodate a wide range of experiences including, but not limited to, physical abuse, sexual abuse, emotional abuse, and neglect, as well as household dysfunction and bullying victimization [2]. In an early treatment study for obesity, Felitti and colleagues discovered an unexpected and strong association between ACEs and adult health later in life [3]. Based on these findings, Felitti et al. conducted the ACE study that was the first to define ACEs as a set of risk factors for subsequent ill health [4]. A range of later studies have consistently chronicled exposure to ACEs to be associated with health risk behaviours and poor health [5–8]. This also applies to unfavourable oral health behaviour and poor oral health [9, 10]. Several direct and indirect pathways explain the link between ACEs and poor health [11]. Adversities that cause prolonged stress may in turn induce epigenetic, neurobiological, and immune response alterations [11–13]. Further, such biological changes are embedded in behavioural changes and may, in synergy with environmental, social, and psychological factors, lead to adoption of health risk behaviour patterns [11]. Health risk behaviours were identified over two decades ago as a mechanism between ACEs and illness later in life [4]. For oral health specifically, this implies that ACEs may be a risk factor for caries development through infrequent toothbrushing, one of many oral health risk behaviours. This study builds on a life course approach to the associations of early life adversity on adult health, suggesting that adverse experiences are major risk factors for the leading causes of illness [14]. We draw upon the ACE-pyramid model [15] to explore how ACEs may have long-term implications for oral health through health risk behaviors.

Adolescence is commonly defined as the period between childhood and adulthood and is characterised by accelerated growth and social role transitions [16]. This period is also known to be critical for oral health due to risk determinants such as a tendency for poor oral hygiene [17] and unhealthy dietary choices [18], which increase the risk of caries [19–21]. As children grow older, they are given ever greater responsibility for daily routines related to oral health; hence, oral hygiene practices may become habitual – or not. The progressive and cumulative nature of caries development makes early establishment of favourable oral hygiene habits critical for oral health in a lifelong perspective [21]. Among young children, it is a parental responsibility to establish and maintain good oral hygiene. However, caries in both primary and permanent teeth is the most common oral condition worldwide, with the highest prevalence among 15–19-year-olds [22].

Most studies on the associations between ACEs and oral health have pooled children and adolescents of all ages [23–28] making it difficult to discern the occurrence and severity of these associations in adolescents. Furthermore, most studies have reported the caries decay component, alone or as part of a DMFT score, as dentine caries lesions and not reported caries lesions limited to enamel. A recent systematic review on dental caries among European adolescents found that enamel caries constituted 50% of all carious lesions [29]. Hence, in adolescents, enamel caries is a considerable part of the caries burden. Thus, enamel caries is important to include as it represents the disease at an early stage as well as an opportunity to prevent development of more severe caries lesions.

Studies investigating the association between ACE and oral health have often relied on parental reports [23, 26, 28, 30]. However, parents may be unaware of adverse experiences occurring outside of the family or may be reluctant to reveal those that have happened at home. Furthermore, parental proxy reports of oral hygiene habits and dental status are not as accurate as adolescent’s self-reports and dental records of clinical measures.

The aim of this cross-sectional study was to explore the association of specific and cumulative ACEs with caries and a common oral health risk behaviour (i.e., toothbrushing frequency) by linking adolescents´ self-reports from the Young-HUNT4 Survey and clinical measures from dental records. Further, we assessed whether any effects were modified by age. Our hypothesis was that ACEs are associated with both non-daily toothbrushing and higher caries experience in adolescents. We also expected a dose-response relationship between ACEs and negative effects on oral health.

## Methods

### Study sample

**Fig. 1:**
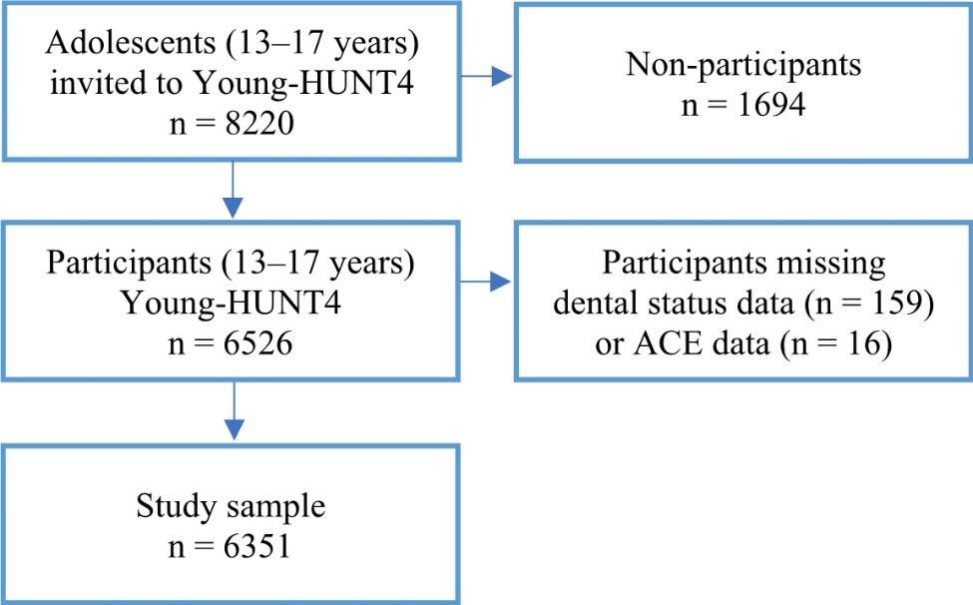
Flowchart for inclusion and exclusion criteria of the study sample

The Young-HUNT Survey is the adolescent segment of the Trøndelag Health Study (the HUNT Study), a large, population-based study in Norway [31, 32]. The Young-HUNT4 Survey is part of the fourth wave of the HUNT Study, conducted between August 2017 and January 2019, to which all adolescents (13–19 years) living in Nord-Trøndelag County were invited. Letters of consent and information were distributed at schools three weeks before the survey. Questionnaires were completed on a tablet during one school hour (40 min) in an exam-like situation with a teacher present in the classroom. Within a month, trained nurses visited the schools for interviews (20 min). In Young-HUNT4, an extra effort was made to reach adolescents not enrolled in the school system (e.g., apprentices or drop-outs). Information letters were sent by mail, inviting these adolescents to participate at apprentice seminars/educational sites, at outreach public services or at HUNT4 field stations. No further reminders were given. In all, 8220 adolescents aged 13–17 years were invited to participate in the Young-HUNT4 survey; of these, 6526 (79.4%) consented to participate. Clinical measures of dental status were extracted from dental health records held by the Public Dental Service (PDS) in Nord-Trøndelag. In Norway, this service provides outreach and free dental health care for all children (0–18 years), and most children ( ∼95%) are enrolled [33]. Dental status was not available for 159 participants, and 16 reported no data on ACEs. The final sample thus consisted of 6351 adolescents: 13–17 years old (Fig. 1).

### Ethical considerations

The Norwegian Data Protection Authority approved the Young-HUNT4 Survey. All participants (aged 16 years and older) and parents (of participants under age 16) gave their informed consent. Consent included access to information on the participants in the Norwegian health and administrative registries and to their medical and dental records [34]. The present study followed all relevant guidelines and regulations and was approved by the Norwegian Regional Committees for Medical and Health Research Ethics (Reference: 97505/REK South East).

### Exposures

The Young-HUNT surveys comprise a questionnaire and a structured interview with questions about somatic and mental health problems, well-being, lifestyle, diet, leisure-time activities, and puberty [31]. From self-report questions in the Young-HUNT4 Survey, we selected eight ACE-related items. Five questions addressing physical abuse, witness to violence and sexual abuse was derived from the UCLA PTSD Reaction Index [35] and adapted to a Norwegian context. Bully victimisation was assessed by four questions derived from validated questionnaires concerning bullying among students [36–38]. In addition, information about parental separation/divorce and parental alcohol problems was used. Table 1 lists the questions and response options for the ACEs in this study.

**Table 1:** Distribution of specific and cumulative adverse childhood experiences by dentine caries experience (DMFT), study sample n=6351

Adverse childhood experience	N (%)	DMFT median (IQR)	DMFT mean (SD)
**Physical abuse, close** (beaten/injured by someone close to me)			
No	5858 (92.2)	1 (0–4)	2.45 (3.26)
Yes^a^	286 (4.5)	2 (0–5)	3.14 (3.34)
Missing	207 (3.3)	2 (0–5)	2.93 (3.78)
**Physical abuse, others** (beaten/injured by others)			
No	5806 (91.4)	1 (0–4)	2.45 (3.24)
Yes^a^	338 (5.3)	2 (0–5)	3.07 (3.67)
Missing	207 (3.3)	2 (0–4)	2.84 (3.75)
**Witness to violence** (seen others violently hurt)			
No	5252 (82.7)	1 (0–4)	2.41 (3.22)
Yes^a^	884 (13.9)	2 (0–4)	2.91 (3.52)
Missing	215 (3.4)	2 (0–4)	2.86 (3.74)
**Sexual abuse, peer** (been subjected to sexually uncomfortable/abusive acts by someone about your age)			
No	5747 (90.5)	1 (0–4)	2.46 (3.25)
Yes^a^	385 (6.1)	2 (0–4)	2.86 (3.49)
Missing	219 (3.5)	1 (0–5)	2.85 (3.77)
**Sexual abuse, adult** (been subjected to sexually uncomfortable/abusive acts by an adult)			
No	5946 (93.6)	1 (0–4)	2.45 (3.25)
Yes^a^	172 (2.7)	3 (1–5)	3.48 (3.66)
Missing	233 (3.7)	2 (0–4)	2.91 (3.71)
**Parental separation/divorce**			
No	4069 (64.1)	1 (0–3)	2.25 (3.16)
Yes	2221 (35.0)	2 (0–4)	2.93 (3.47)
Missing	61 (1.0)	3 (1–5)	3.31 (3.33)
**Parental alcohol problems** (ever seen either of your parents intoxicated)			
No	5982 (94.2)	1 (0–4)	2.44 (3.22)
Yes^b^	248 (3.9)	3 (1–6)	3.83 (4.16)
Missing	121 (1.9)	1 (0–4)	2.70 (3.68)
**Bully victimization** ^c^			
No	5056 (79.6)	1 (0–4)	2.46 (3.23)
Yes	1001 (15.8)	1 (0–4)	2.67 (3.55)
Missing	294 (4.6)	1 (0–4)	2.58 (3.39)
**Number of ACEs**			
0	2691 (42.4)	1 (0–3)	2.13 (3.02)
1	2014 (31.7)	1 (0–4)	2.58 (3.36)
2	704 (11.1)	2 (0–4)	2.93 (3.45)
3	249 (3.9)	2 (0–5)	2.98 (3.49)
≥4	230 (3.6)	2 (0–6)	3.50 (3.90)
Missing information on one or more ACEs	463 (7.3)	2 (0–4)	2.83 (3.47)

N: Number of participants; DMFT: Decayed, Missing, and Filled Teeth; IQR: Interquartile range; SD: Standard deviation; ACE: Adverse childhood experience

^a^Once/several times

^b^A few times a month/a few times a week

^c^Bully victimization was assessed by four questions: “I have been made fun of, teased in a hurtful way by peers, or someone has said ugly things to me”; ”I have been bullied, hit, got my hair pulled, kicked or attacked by peers”; “I have been isolated by peers and I am not allowed to join them”; “I have received unpleasant messages or pictures on my cell phone or via internet”. The response options were identical in all four types of bully victimization (never, 1–3 times a month, once a week, 2–4 times a week). We defined experiencing one type of bullying once a week or experiencing two types of bullying 1–3 times a month as an ACE.

To evaluate the dose-response effect of exposure to multiple adversities, the self-reported specific ACEs were summed to determine cumulative ACE exposure (range, 0–8). A higher score reflects a higher number of adversities experienced by the adolescents.

### Outcomes

Toothbrushing frequency was the measure used to assess oral hygiene. The questionnaire item was “How often do you brush your teeth?”. We dichotomized the response options into daily toothbrushing (twice a day or more/once a day) and non-daily toothbrushing (occasionally [not every day]/never).

Caries data was extracted from existing dental health records held by the PDS in Nord-Trøndelag County. The Decayed, Missing, and Filled Teeth (DMFT) index, an aggregate of current and past caries in permanent teeth (i.e., dentine caries decay, restorations, and teeth missing due to caries) [39], was used to assess caries experience at dentine level. Enamel caries was used as a separate measure. A five-graded caries diagnosis system was adopted, with grades 1 and 2 representing caries limited to enamel and grades 3–5 representing caries also involving dentine [40]. The diagnoses were based on both clinical and radiographic (bitewing) findings [41]. Caries data were gathered from annual dental health summaries in 2018 to represent the same period as participation in the Young-HUNT4 Survey (data collection: August 2017–January 2019). If an individual did not get a dental exam in 2018, the dental health summary from 2017 or 2019 was used.

The five-graded caries diagnostic system used by the PDS is taught at the Norwegian dental faculties and integrated in the dental health record system (Opus Dental). Since 2013, the PDS in Nord-Trøndelag has organized one-day training seminars for dentists and dental hygienists. These seminars have included radiograph-based caries diagnostic training (i.e., 19 bitewing sets) and case discussion. In addition, prior to and in conjunction with the Young-HUNT4 data collection, dentists (n = 38) and dental hygienists (n = 15) from the PDS in Nord-Trøndelag attended a one-day caries diagnostic training seminar in 2017. The program featured a theoretical section on caries classification and a training session in which participants annotated bitewing radiographs in smaller groups. The bitewing annotation results were discussed in plenary, but no reliability scores were computed.

### Covariates

Potential confounding factors were retrieved from the questionnaires and interviews in the Young-HUNT4 Survey and included demographic and socioeconomic factors: Age (continuous), sex (male/female), biological parents’ birth country (categorized as both/one/none of the parents being Nordic born), parental employment, family economy, and living arrangements [42–44]. Parental employment status was self-reported by responding to the question: “Is your father/mother or foster father/mother or other guardian in paid work?” (no/yes). Self-perceived family economy was assessed by the question: “How well off do you think your family is compared to most others?” The responses were dichotomized into adequate (better financial situation/about the same as most others) and poor (worse financial situation). The participants’ living arrangements were captured by the question “Who do you usually live with?” categorized as both parents/both parents but shared/one of the parents/not living with parents (i.e., living with other caregivers, at an institution, in a dorm, or alone without caregivers).

### Statistical analyses

All statistical analyses were performed using Stata v17 (Stata Corp., College Station, Texas, USA). The significance level was set to a p-value below 0.05 for all tests. The associations between ACEs and toothbrushing frequency were evaluated using logistic regression models. The odds ratios (ORs) and 95% confidence intervals (CIs) were computed after adjustment for potential confounders including age, sex, biological parents’ birth country, parental employment, family economy, and living arrangements. Further, associations between ACEs and dentine caries experience (DMFT) were evaluated using negative binomial regression models adjusted for the same confounders. Negative binominal regression models were used due to the skewed distribution of the outcome count variable (DMFT). Incident rate ratios (IRRs) with 95% confidence intervals (CIs) were calculated [45]. In a cross-sectional design, the IRR of the negative binomial regression analysis can be regarded as a ratio of means (RM), where the mean number (count) is increasing if IRR > 1 and decreasing if IRR < 1 [46]. In the present study, the result was interpreted as an (IRR-1)*100% change in mean DMFT (i.e., number of teeth with dentine caries experience) relative to the exposure variable. Similarly, negative binominal regression models were used to assess the associations between ACEs and enamel caries.

The likelihood ratio test was used to assess potential effect modification by age (13–15 vs. 16–17 years) on the associations of ACEs with toothbrushing frequency, dentine caries experience and enamel caries.

## Results

This study is based on a general adolescent population of 6351 participants from the Young-HUNT4 Survey. The mean age of the population was 15.51 (SD 1.46) and 51.2% were females. The mean DMFT (i.e., mean number of permanent teeth with dentine caries experience) of the population was 2.50 (SD 3.29). The 16–17-year-olds presented with a higher caries experience compared to the 13–15-year-olds. Adolescents reporting daily toothbrushing experienced less caries compared to those reporting non-daily toothbrushing, Table 2.

**Table 2:** Characteristics of the study sample by dentine caries experience (DMFT)

Variables	N (%)	DMFT median (IQR)	DMFT mean (SD)
**Total**	6351	1 (0–4)	2.50 (3.29)
**Sex**			
Female	3249 (51.2)	1 (0–4)	2.60 (3.37)
Male	3102 (48.8)	1 (0–4)	2.39 (3.19)
**Age**, mean (SD)	15.51 (1.46)		
13–15 years	3629 (57.1)	1 (0–3)	1.88 (2.66)
16–17 years	2722 (42.9)	2 (0–5)	3.31 (3.82)
**Nordic birth country parents**			
Both parents	5590 (88.0)	1 (0–4)	2.48 (3.28)
One parent^a^	331 (5.2)	1 (0–3)	2.31 (3.03)
None of the parents^b^	401 (6.3)	2 (0–4)	2.95 (3.57)
Unknown/missing	29 (0.5)	1 (0–5)	2.48 (3.08)
**Family economy**			
Better/same financial situation as most others	5813 (91.5)	1 (0–4)	2.46 (3.27)
Worse financial situation	463 (7.3)	2 (0–4)	2.86 (3.45)
Unknown/missing	75 (1.2)	2 (0–6)	3.28 (3.54)
**Parental employment**			
Both parents in paid work	4636 (73.0)	1 (0–3)	2.26 (3.07)
One parent in paid work^c^	1205 (19.0)	2 (0–4)	2.87 (3.64)
None of the parents in paid work^d^	198 (3.1)	3 (0–6)	3.88 (4.08)
Unknown/missing	312 (4.9)	2 (0–6)	3.60 (3.84)
**Living arrangements**			
Both parents	3880 (61.1)	1 (0–3)	2.20 (3.09)
Both parents, but shared	1110 (17.5)	2 (0–4)	2.56 (3.26)
One of the parents	804 (12.7)	2 (0–5)	3.16 (3.56)
Not living with parents	260 (4.1)	2 (0–5)	3.30 (3.86)
Unknown/missing	297 (4.7)	2 (0–6)	3.60 (3.88)
**Toothbrushing frequency**			
Daily	5910 (93.1)	1 (0–4)	2.39 (3.16)
Non-daily	406 (6.4)	3 (1–6)	4.18 (4.48)
Missing	35 (0.6)	0 (0–1)	1.11 (1.92)

N: Number of participants; DMFT: Decayed, Missing, and Filled Teeth; IQR: Interquartile range; SD: Standard deviation

^a^Included 56 participants with a missing answer for one of the parents

^b^Included 31 participants with a missing answer for one of the parents

^c^Included 141 participants with a missing answer for one of the parents

^d^Included 42 participants with a missing answer for one of the parents

Adolescents exposed to any specific adversity displayed a higher mean DMFT compared to those not exposed to that specific ACE. Furthermore, the mean DMFT score gradually increased with increasing number of adversities, Table 1.

Exposure to any of the five specific ACEs – physical abuse by others, witness to violence, sexual abuse by a peer, parental separation/divorce, or bully victimization – was associated with non-daily toothbrushing when compared with those with no exposure to the given ACE. For each additional adversity experienced, there was a 30% higher likelihood of reporting non-daily toothbrushing (OR 1.30, 95% CI 1.19–1.42). Further, there was evidence of effect modification by age with bully victimization (P_interaction_ = 0.014); in particular, bully victimization was associated with non-daily toothbrushing among 16–17-year-olds (OR 2.59, 95% CI 1.80–3.72), Table 3.

**Table 3:** Associations between adverse childhood experiences and non-daily toothbrushing in adolescents

ACE^a^		MAIN ANALYSIS	SUBGROUP ANALYSIS		
	N	OR^b^ (95% CI)	13–15 yrs OR^b^ (95% CI)	16–17 yrs OR^b^ (95% CI)	P_interaction_
Physical abuse, close	5785	1.42 (0.92–2.20)	1.48 (0.76–2.88)	1.45 (0.81–2.59)	0.727
Physical abuse, others	5786	2.19 (1.55–3.11)	2.01 (1.16–3.48)	2.42 (1.52–3.84)	0.779
Witness to violence	5780	1.54 (1.18–2.01)	1.55 (1.03–2.33)	1.61 (1.12–2.30)	0.932
Sexual abuse, peer	5772	2.09 (1.40–3.11)	2.21 (1.18–4.13)	2.14 (1.26–3.62)	0.594
Sexual abuse, adult	5759	1.01 (0.52–1.97)	1.08 (0.38–3.11)	1.00 (0.42–2.41)	0.719
Parental separation/divorce	5889	1.65 (1.09–2.50)	2.37 (1.32–4.26)	1.38 (0.78–2.44)	0.111
Parental alcohol abuse	5855	1.53 (0.97–2.43)	1.99 (0.93–4.26)	1.29 (0.73–2.29)	0.314
Bully victimization	5702	1.86 (1.44–2.40)	1.36 (0.94–1.97)	2.59 (1.80–3.72)	0.014
**Per ACE increase**	5559	1.30 (1.19–1.42)	1.29 (1.13–1.47)	1.32 (1.18–1.48)	0.803

ACE: Adverse childhood experience; N: Number of participants; OR: Odds ratio; CI: Confidence interval

^a^The reference for each specific ACE was no history of exposure to the given ACE

^b^Adjusted for age (continuous), sex (male/female), Nordic birth country parents, family economy, parental employment status and living arrangements

The adverse experiences of parental separation/divorce and parental alcohol problems were both associated with increased dentine caries experience when compared with no exposure to the specific ACE. Furthermore, the effect sizes of parental separation/divorce, parental alcohol problems, witness to violence, sexual abuse by an adult, and bully victimization were more predominant among 16–17-year-olds. We also found evidence of effect modification by age for bully victimization (P_interaction_ < 0.001), and the effect primarily occurring among 16–17-year-olds (IRR 1.30, 95% CI 1.14–1.50), Table 4.

**Table 4:** Associations between adverse childhood experiences and dentine caries experience (DMFT)

ACE^a^		MAIN ANALYSIS	SUBGROUP ANALYSIS		
	N	IRR^b^ (95% CI)	13–15 yrs IRR^b^ (95% CI)	16–17 yrs IRR^b^ (95% CI)	P_interaction_
Physical abuse, close	5790	1.14 (0.97–1.35)	1.03 (0.80–1.33)	1.22 (0.99–1.51)	0.323
Physical abuse, others	5791	1.12 (0.96–1.30)	1.13 (0.90–1.42)	1.11 (0.91–1.36)	0.970
Witness to violence	5785	1.07 (0.97–1.18)	1.00 (0.86–1.16)	1.15 (1.01–1.30)	0.128
Sexual abuse, peer	5777	1.01 (0.88–1.17)	1.03 (0.81–1.30)	0.99 (0.83–1.19)	0.758
Sexual abuse, adult	5764	1.19 (0.97–1.46)	1.05 (0.75–1.47)	1.29 (1.00-1.67)	0.418
Parental separation/divorce	5912	1.27 (1.10–1.48)	1.15 (0.92–1.43)	1.46 (1.19–1.80)	0.885
Parental alcohol abuse	5861	1.32 (1.11–1.56)	1.21 (0.89–1.63)	1.37 (1.12–1.68)	0.436
Bully victimization	5707	1.08 (0.98–1.19)	0.94 (0.82–1.07)	1.30 (1.14–1.50)	< 0.001
**Per ACE increase**	5564	1.06 (1.02–1.09)	1.00 (0.95–1.06)	1.10 (1.05–1.16)	0.026

ACE: Adverse childhood experience; N: Number of participants; IRR: Incident rate ratio; CI: Confidence interval

^a^The reference for each specific ACE was no history of exposure to the given ACE

^b^Adjusted for age (continuous), sex (male/female), Nordic birth country parents, family economy, parental employment status, and living arrangements

We observed a positive linear association between increasing number of reported adversities and higher dentine caries experience, with a 6% increase in mean DMFT for every additional adversity (IRR 1.06, 95% CI 1.02–1.09). Also, there was evidence of modification by age (P_interaction_ = 0.026) and the effect was predominantly among the 16–17-year-olds (IRR 1.10, 95% CI 1.05–1.16), Table 4.

We found an association between several specific ACEs and a higher enamel caries experience among exposed adolescents compared to those with no exposure to the given ACE. Further, a positive linear association between increasing number of reported adversities and a higher mean number of teeth with enamel lesions was observed (IRR 1.07, 95% CI 1.03–1.11). The likelihood ratio test yielded no evidence of modification by age in the association between specific ACEs and enamel caries, Table 5.

**Table 5:** Associations between adverse childhood experiences and enamel caries

ACE^a^	N	MAIN ANALYSIS	SUBGROUP ANALYSIS		
		IRR^b^ (95% CI)	13–15 yrs IRR^b^ (95% CI)	16–17 yrs IRR^b^ (95% CI)	P_interaction_
Physical abuse, close	5790	1.17 (0.99–1.38)	1.09 (0.85–1.41)	1.24 (1.00-1.53)	0.773
Physical abuse, others	5791	1.19 (1.03–1.38)	1.21 (0.96–1.51)	1.19 (0.98–1.44)	0.762
Witness to violence	5785	1.14 (1.03–1.26)	1.13 (0.98–1.32)	1.14 (1.00-1.29)	0.889
Sexual abuse, peer	5777	1.08 (0.93–1.24)	1.22 (0.97–1.54)	0.99 (0.83–1.19)	0.083
Sexual abuse, adult	5764	1.07 (0.87–1.32)	1.09 (0.78–1.54)	1.09 (0.84–1.42)	0.657
Parental separation/divorce	5912	1.25 (1.08–1.45)	1.26 (1.02–1.55)	1.26 (1.03–1.55)	0.371
Parental alcohol abuse	5861	1.26 (1.06–1.50)	1.33 (0.99–1.80)	1.21 (0.99–1.49)	0.510
Bully victimization	5707	1.12 (1.02–1.23)	1.05 (0.92–1.19)	1.22 (1.06–1.40)	0.128
**Per ACE increase**	5564	1.07 (1.03–1.11)	1.06 (1.01–1.12)	1.08 (1.03–1.13)	0.693

ACE: Adverse childhood experience; N: Number of participants; IRR: Incident rate ratio; CI: Confidence interval

^a^The reference for each specific ACE was no history of exposure to the given ACE

^b^Adjusted for age (continuous), sex (male/female), Nordic birth country parents, family economy, parental employment status and living arrangements

## Discussion

In this population-based cross-sectional study of 6351 adolescents aged 13–17 years, we found that adolescents with a history of adverse experiences were more likely to report infrequent toothbrushing and have a higher dentine caries experience compared to peers with no exposure to adverse experiences. This association, however, occurred for some, but not all, ACEs. Further, there was evidence of a dose-response relationship; each additional adversity was associated with a higher likelihood of non-daily toothbrushing and an increase in both mean DMFT and mean number of teeth with enamel caries.

We included toothbrushing frequency as a measure of oral health risk behaviour, as toothbrushing with fluoride toothpaste is considered the first-line prophylaxis for caries [47–49]. Our results suggest an association between ACEs and non-daily toothbrushing. These findings are in line with other paediatric studies reporting an association between ACEs and poor oral hygiene, as measured by severity of gingival inflammation or dental plaque [25, 26, 50]. In contrast, Folayan et al. observed no association between adverse experiences and oral hygiene among Nigerian children and adolescents [51].

Further, the study findings are in line with the results of other studies exploring the presence of caries in children exposed to adversities [24, 25, 27, 52]. However, we also found evidence of an effect modification by age with dentine caries experience being more predominant in the older, 16–17-year-old group than among the 13–15-year-olds. In fact, our results suggest no association between ACEs and dentine caries experience in 13–15-year-olds, which is in line with a suburban Nigerian study among 6–16-year-olds (96% of the study sample was 11–16 years) by Folayan et al. [51]. In agreement with Folayan, we hypothesize this could partly be explained by the fact that as adolescents grow older, ACEs have more time to suffer an impact on oral health, in a “wear and tear” manner. Younger adolescents have many newly erupted permanent teeth, and since caries is a cumulative disease, the impact of ACEs on dentine caries experience may be less detectable in these ages. Hence, including enamel caries, which reflect initial caries activity, may provide a better understanding of the association between ACEs and caries. We did find an association between increasing number of reported adversities and higher enamel caries experience among both younger and older adolescents, revealing an impact in the earlier phase in caries development.

Kvist et al. observed that bully victimization had a surprisingly great impact on self-reported oral health. [53]. Their finding was later corroborated with clinical measures of caries (i.e., DMFT) in a study from Brazil [54], which is in line with our findings – where reporting bully victimization was associated with a 30% higher mean DMFT compared to non-victimized peers among 16-17-year-olds. We also observed a two-fold higher likelihood of non-daily toothbrushing among 16–17-year-old victims of bullying. In other words, bully victimization may have a considerable impact on oral health.

Several mechanisms linking adversities to poor health outcomes later in life have been proposed, including the adoption of health risk behaviours, a leading cause of morbidity [4, 11]. In the context of oral health risk behaviours, it has been suggested that the prolonged stress induced by adversities may both occupy and deplete the resources necessary for self-care [55], or leave the individual less motivated to pay attention to oral self-care [56]. Further, prolonged exposure to stress is linked to learning difficulties [11, 57]. Chronic stress may be all-consuming, potentially occupying the individual’s resources to feel, reflect, and be mentally present, resulting in the loss of valuable learning opportunities, such as learning and adapting to oral hygiene practises. In addition, it has been suggested that vulnerable family settings may reduce the caregiver’s ability and capacity to provide responsive care, including the establishment and follow-up of oral hygiene habits [23].

As ACEs pose a major risk for developing illness in adulthood through the adoption of health risk behaviours, identifying adolescents at risk in the dental setting is important for preventing an unfortunate oral health trajectory. Even though health professionals are required by law to report concerns about child abuse or neglect, there are several barriers asking about and reporting these concerns [9, 58]. Continuously encouraging dental practitioners to ask their patients about adverse experiences is therefore important for identifying vulnerable individuals. Moreover, given that a history adverse childhood experiences is associated with a higher likelihood of learning difficulties [57], dental health professionals should recognize that these adolescents may require individualized education and support on oral hygiene practises. In addition, experiencing adversities may manifest in a lack of trust [59, 60]. In a patient-dentist relationship, it is therefore important to be aware that addressing poor oral hygiene habits in vulnerable adolescents requires an empathic approach. A safe and non-judgmental environment can be crucial for receiving care.

The population-based approach, along with the self-reports of the adolescents and clinical data retrieved from dental records, are major strengths of the present study. However, the results should be interpreted in the context of certain limitations. First, using data for dental health records held by the PDS involve many examiners. Despite PDS-organized training sessions, the calibration was not optimal and examiners’ reliability values were omitted. Secondly, the Young-HUNT4 Survey did not address all types of childhood trauma nor all potential confounding factors. Further, we did not consider the duration, timing, and synergistic effects of the studied adversities. Another issue refers to the self-reporting of previously experienced adversities, which may be subject to recall and social desirability biases. Delayed disclosure of childhood sexual abuse is common, and long delays are typical [61, 62]. Hence, the associations of this particular ACE with poor oral health parameters may be underestimated. In addition, dropout analyses of previous HUNT surveys in adults found a lower socioeconomic status and poorer health among the non-participants compared with survey participants [63]. There is reason to believe that this may also occur among non-participants in an adolescent population and may have affected our results. Finally, because of the cross-sectional design, we cannot determine temporal associations of ACEs with toothbrushing frequency and caries.

## Conclusions

ACEs may have lasting effects on oral health and oral health behaviours. This study found that several specific ACEs were associated with non-daily toothbrushing and a higher mean number of teeth with caries experience among Norwegian adolescents in the Young-HUNT4 Survey. Further, dose-response relationships of increasing numbers of adversities with higher caries experience and with infrequent toothbrushing were found.

## Data Availability

Provided approval from HUNT Research center, sharing of data from the present investigation will be supported by the corresponding author upon reasonable request. For more information see: www.ntnu.edu/hunt/data. Inquiries regarding access to data is directed to: kontakt@hunt.ntnu.no.

## Abbreviations

ACE: Adverse childhood experience
CI: Confidence interval
DMFT: Decayed, Missing, and Filled Teeth
HUNT: The Trøndelag Health Study
IQR: Interquartile range
IRR: Incident rate ratio
OR: Odds ratio
PDS: Public Dental Service
SD: Standard deviation
Young-HUNT4 Survey: Adolescents in the fourth wave of the HUNT Study
